# Immunotherapeutic role of Ag85B as an adjunct to antituberculous chemotherapy

**DOI:** 10.1186/1476-8518-9-4

**Published:** 2011-06-26

**Authors:** Javaid A Sheikh, Gopal K Khuller, Indu Verma

**Affiliations:** 1 Department of Biochemistry, Postgraduate Institute of Medical Education and Research, Chandigarh 160012, India

## Abstract

**Background:**

Immunotherapy to enhance the efficiency of the immune response in tuberculosis patients and to eliminate the persisters could be an additional valuable strategy to complement anti-mycobacterial chemotherapy. This study was designed to assess the immunotherapeutic potential of Ag85B as an adjunct to chemotherapy and its effect against active and persister bacteria left after therapy in mouse model of tuberculosis.

**Methods:**

6-8 week old female Balb/c mice were infected with *Mycobacterium tuberculosis *and treated with chemotherapy or immunotherapy. Protective efficacy was measured in terms of bacterial counts in lungs and spleen. Immune correlates of protection in terms of Th1 and Th2 cytokines were measured by ELISA.

**Results:**

Therapeutic effect of Ag85B was found to be comparable to that of short term dosage of antituberculous drugs (ATDs). The therapeutic effect of ATDs was augmented by the simultaneous treatment with rAg85B and moreover therapy with this protein allowed us to reduce ATD dosage. This therapy was found to be effective even in case of drug persisters. The levels of antigen specific IFNγ and IL-12 were significantly increased after immunotherapy as compared to the basal levels; moreover antigen specific IL-4 levels were depressed on immunotherapy with Ag85B.

**Conclusion:**

We demonstrated in this study that the new combination approach using immunotherapy and concurrent chemotherapy should offer several improvements over the existing regimens to treat tuberculosis. The therapeutic effect is associated not only with initiating a Th1 response but also with switching the insufficient Th2 immune status to the more protective Th1 response.

## Background

Major obstacle in control of tuberculosis being poor patient compliance with the protracted regimen in areas with limited resources which may lead to relapse of active disease, transmission of infection and development of drug resistant strains [1]. In such circumstances, immunotherapy to enhance the efficiency of the immune response in *M. tuberculosis *infected patients could be an additional valuable strategy to complement anti-bacterial chemotherapy. Even immunotherapy might shorten the duration of treatment for drug-susceptible tuberculosis, thereby reducing the cost and increasing treatment completion rates or might increase the cure rates in case of MDR tuberculosis. In last decade, various nonspecific or antigen specific immunological agents have been used either alone or as an adjunct to chemotherapeutic regimen with variable success [2,3] such as, DNA plasmids [4,5], detoxified *M. tuberculosis *extract in liposomes (RUTI) [6], *Mycobacterium vaccae *[7], cytokines [8], Immunoglobulins [9], mycobacterial antigens [10] etc, to name a few. Thus, considering the advancement in the field and keeping in view the potential clinical aims, further research on the concept of immunotherapy, or as an adjunct to chemotherapy of tuberculosis, seems to be valuable.

Ag85B, a 30 kDa fibronectin-binding protein with mycolyltransferase activity, is a major protein secreted by all mycobacterium species and belongs to the Ag85 family. Ag85B is highly immunogenic, as shown by the easy detection of specific humoral and cell-mediated immune responses both in latently and actively infected TB patients [11,12]. It has also been shown to induce a strong Th1-type immune response in mice as well as in humans. Several studies have shown a significant protective effect in the lungs of mice immunized with Ag85B [13-16], whereas a few contradictory reports on the efficacy of Ag85B protein vaccination have also been reported [17-19]. Recently we reported the immunotherapeutic effect of Ag85AB complex as a whole in mouse model [10].

Analysis of the immunological mechanism in various models suggest that the induction of Th1 immune response including antigen-specific CD8^+^/CD4^-^/CD44^high ^memory type cytotoxic T cells producing IFNγ, is required for TB therapeutic vaccine efficacy in humans as well [20-22]. Since DNA vaccines are known to establish cellular immune responses, including cytotoxic T-lymphocyte (CTL) and Th1 responses, much interest is being given to them. Their prophylactic behaviour [15,17,23-25] was found to be effective at limiting the growth of *M. tuberculosis *in mice, but their therapeutic use has been largely controversial [2].

Present study was carried out to better investigate the immunotherapeutic effect of Ag85B protein and a DNA vaccine based on this protein in mouse model of tuberculosis. Its adjunctive immunotherapeutic effect with simultaneous conventional chemotherapy and moreover its effect on the persisters left after short-term, non-sterilizing chemotherapy was also investigated. This was done with the main objective of understanding the immunological mechanisms involved in the therapeutic anti-TB immune response.

## Results

### Therapeutic effect of the Ag85B protein and its DNA vaccine

**Figure 1:**
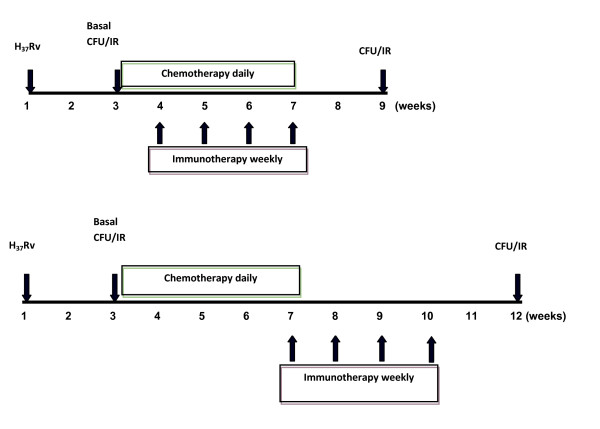
**Time line representation of the experimental design**. Schematic time line representation of the experimental design to analyze the immunotherapeutic effect of adjunctive immunotherapy (upper) and the effect of immunotherapy on the persister bacteria (lower). Animals treated with immunotherapy or chemotherapy alone, were treated along same timeline as shown in upper panel. (CFU: Colony Forming Units; IR: Immune Responses)

**Figure 2:**
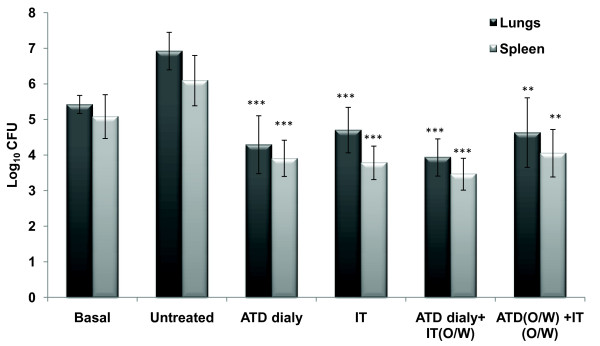
**Increased protection in animals as depicted by numbers of viable CFUs of *M. tuberculosis *H_37_Rv in the lungs of *M. tuberculosis *H_37_Rv infected animals receiving adjunctive immunotherapy**. Two weeks post chemotherapy/immunotherapy, lungs and spleen from treated and untreated *M. tuberculosis *H_37_Rv infected animals were isolated and cultured on Middlebrook 7H11 agar plates. Results are expressed as mean log_10 _CFUs ± standard deviation of 5 animals per group tested individually. Statistical analysis of the results was carried out by Student's *t *test. ***p< 0.001, **p< 0.01 compared to untreated animals. Basal level represents CFU after two weeks of infection. (ATD: Antituberculous Drugs; IT: Immunotherapy; O/W: Once Weekly)

Mice infected with the *M. tuberculosis *H_37_Rv strain were treated with either rAg85B protein or Ag85B-DNA (Figure 1), and the therapeutic effects were expressed as the bacterial load in the spleen and lung (Figure 2 & Table 1). Compared with the adjuvant immunized control group, rAg85B significantly reduced the bacterial numbers in the spleen and lung (*p*< 0.001), and was equally effective to that of 'short term' chemotherapy. The adjunctive effect of immunotherapy and chemotherapy was much more pronounced as compared to adjuvant immunized control group (*p*< 0.001) showing effective cumulative effect on bacterial eradication (Table 1). Although the effect was not additive as marginal decrease in CFU count was not statistically significant when compared to ATD or IT alone. However no significant reduction in CFUs with Ag85B-DNA immunotherapy was observed (data not shown). Further efforts were made to mimic the process of chemotherapy dosage reduction by delivery of drug dosage just once a week with simultaneous immunotherapy and a significant reduction in CFU was observed as compared to that of control (*p*< 0.01). These findings suggest that immunotherapy could be effectively used as an adjunct to chemotherapy and drug dosage can be considerably reduced to once a week with concomitant immunotherapy, as the CFU reduction was same as that of the group where ATD was given daily (Figure 2).

**Table 1:** Bacterial load in terms of CFU in lung and spleen of infected animals after immunotherapy and short-term chemotherapy

Groups	No. of CFU(× 10^5^) in					
	
	Lung	± 95% CI	P value	Spleen	± 95% CI	P value
**Basal**	2.66 ± 0.254	0.22 (5.20-5.64)		1.22 ± 0.616	0.54 (4.54-5.62)	

**Untreated**	83.72 ± 0.527	0.46 (6.46-7.38)		12.5 ± 0.707	0.62 (5.47-6.71)	

**ATD dialy**	0.196 ± 0.814	0.71 (3.58-5.00)	0.0003 *	0.080 ± 0.509	0.45 (3.45-4.35)	0.0005 *

**IT**	0.507 ± 0.639	0.56 (4.14-5.26)	0.0003 *	0.061 ± 0.47	0.41 (3.37-4.19)	0.0003 *

**ATD dialy+ IT(o/w)**	0.086 ± 0.523	0.46 (3.47-4.39)	0.0001 *	0.029 ± 0.448	0.39 (3.07-3.85)	0.0001 *

**ATD(o/w) +IT (o/w)**	0.435 ± 0.978	0.86 (3.77-5.49)	0.0017 *	0.114 ± 0.667	0.58 (3.47-4.63)	0.0016 *

**Untreated (2 months)**	147 ± 0.551	0.48 (6.68-7.64)		15.6 ± 0.028	0.02 (6.17-6.21)	

**ATD dialy 1 month + 1 month adjuvant immunized**	1.29 ± 0.315	0.28 (4.83-5.39)		2.70 ± 0.99	0.87 (4.56-6.3)	

**ATD dialy (1 month) + IT next month (o/w)**	0.31 ± 0.319	0.28 (4.21-4.77)	0.0001 $ 0.014 #	0.088 ± 0.44	0.39 (3.55-4.33)	0.0001 $ 0.015 #

* With respect to Untreated

$ With respect to group Untreated (2 months)

# With respect to group ATD daily 1 month + 1 month adjuvant immunized

CFU: colony Forming Units; ATD: Anti Tuberculous Drugs; IT: Immunotherapy; o/w: Once Weekly;

### Immunotherapeutic effect on persister bacteria

**Figure 3:**
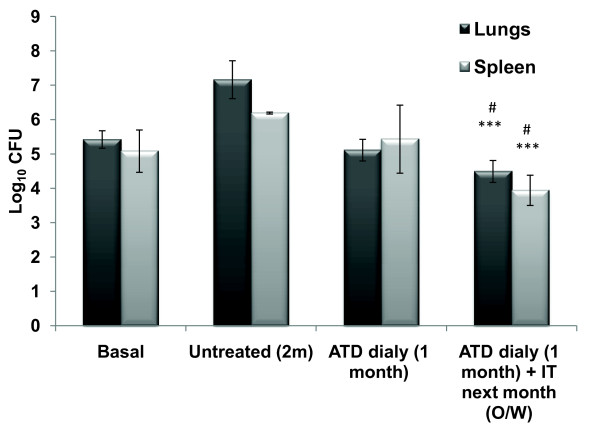
**Effect of immunotherapy on persisters in terms of Log_10 _CFUs of Mtb at two weeks post treatment in the lungs and spleen of Mtb infected animals**. Two weeks post challenge with Mtb, mice were treated with ATD for 4 weeks and then 4 doses of rAg85B weekly. Two weeks post treatment lungs and spleen from *M. tuberculosis *H_37_Rv infected animals were isolated and cultured on Middlebrook 7H11 agar plates. Results are expressed as mean log_10 _CFUs ± standard deviation of 5 animals per group tested individually. Statistical analysis of the results was carried out by Student's *t *test. ***p< 0.001, compared to animals that were left untreated during the treatment period of eight weeks. #*p*< 0.05, compared to animals receiving ATD for four weeks. (ATD: Antituberculous Drugs; IT: Immunotherapy; O/W: Once Weekly)

After the completion of conventional chemotherapy, the bacteria that survive are usually persisters and in order to check the efficacy of current immunotherapy on those persisters, mice were administered a four week dose of chemotherapy to eliminate the drug susceptible bacteria and then were subjected to immunotherapy. Significant reduction in bacterial CFUs was observed as compared to group where mice were adjuvant immunized for 1 month after chemotherapy; implying that immunotherapy with Ag85B protein was effective in inhibiting the persisters to regrow, that could resist the 'short term' chemotherapy (Figure 3).

### Cytokine profile after Immunotherapy with rAg85B

**Figure 4:**
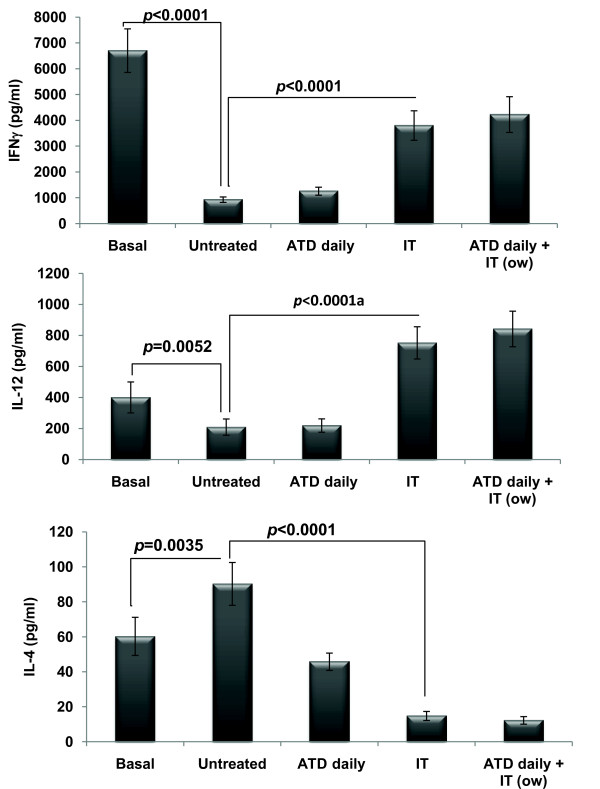
**Cytokine secretion by PBMCs isolated from untreated/treated tuberculous mice**. Two weeks after the treatment, PBMCs were obtained from the untreated/treated tuberculous mice (5 animals per group) and *in vitro *stimulated with or without rAg85B in quintuplicates. Supernatants were collected from the cultures, and the levels of respective cytokines were measured by ELISA assay. The results are expressed as mean ± standard deviation of five OD values after deducting the values of unstimulated wells. Statistical analysis of the results was carried out by Student's *t *test. The basal levels of cytokines were estimated two weeks after infection with *M. tuberculosis*, just before the treatment. (ATD: Antituberculous Drugs; IT: Immunotherapy; O/W: Once Weekly)

The levels of Th1 (IFNγ and IL-12) and Th2 (IL-4) cytokines were monitored before and after immunotherapy. The levels of antigen specific IFNγ and IL-12 were significantly increased after immunotherapy as compared to that of untreated group. Moreover, antigen specific IL-4 levels declined upon immunotherapy with Ag85B (Figure 4). On the contrary there was no significant level of antigen induced cytokines in the mice receiving immunotherapy with plasmid expressing Ag85B (data not shown).

## Discussion

Immunotherapy that modulates or enhances the host immune response to *M. tuberculosis *has proven to be an effective method for treatment of tuberculosis in mice [26,27]. The long treatment course, along with the side effects, often results in treatment failure. These limitations together with the increasing incidence of drug resistant strains and co infection with HIV point to an urgent need for additional immunotherapeutic regimens [28,29].

Effective antimycobacterial immunity is presumed to be due to Th1 response, which is dominated by antigen-specific T lymphocytes that produce IFNγ and are cytotoxic towards infected cells [20-22]. Th2 response characterized by IL-4 production, which is predominant during infection with *M. tuberculosis*, has been reported to be non-protective in TB [20,30,31]. A shift in the balance towards Th1 response may be beneficial, laying down a criterion for the selection of the antigens to be protective in case of tuberculosis. Their prophylactic behaviour being the guiding line for their probable therapeutic use on basis of the principle 'diamond cuts diamond' on the contrary to that of 'adding fuel to the fire'.

Compared to antituberculosis drugs, the natural immune response has a minor impact on mycobacterial elimination during the early phase of therapy [32]. It is expected that immune modulation by immunotherapy will lead to significant increase in the cure rates. The present study involved the evaluation of immunotherapeutic effect of rAg85B and a DNA vaccine expressing this protein. Immunotherapy with rAg85B led to significant decrease in bacterial load in both lungs and spleen which was comparable to that of short term chemotherapy (Figure 2). A two log reduction observed in the lungs of mice receiving immunotherapy with rAg85B indicated it as an effective measure, as suggested by Orme *et al *[33] according to which measures reducing pulmonary bacterial loads by 0.7 logs are considered to be effective. On the contrary, no such protection was observed in case of pVAX85. This absence of protection in case of DNA vaccination is inconsistent with the results from the published literature [26] but still, though hard to justify, has supporting evidence [2]. Further, Ag85B immunotherapy, combined with short term chemotherapy over 4 weeks showed a stronger therapeutic effect than chemotherapy or immunotherapy alone when compared to that of adjuvant immunized control group (Table 1). Protective effect was still significant even when the chemotherapeutic dose was reduced to once weekly instead of daily, thus suggesting the possibility that immunotherapy including Ag85B combined with chemotherapy might shorten the period of conventional chemotherapy (Figure 2). However, a time and antigen titration approach may determine an optimal combined regimen sufficient to confer superior therapeutic activity.

In this study a short dose of non-sterilizing chemotherapy was administered so that only drug susceptible bacteria are eliminated. The persisters left out were treated with rAg85B immunotherapy and a significant reduction in persister bacterial count was observed (Figure 3). These findings suggest utility of Ag85B based immunotherapy against regrowth of persisters which are the major cause of protracted drug treatment and even relapse of active disease. This role of immunotherapy seems to be particularly important because the most organisms being extracellular during the initial phase of therapy are vulnerable to drugs but immunotherapy stimulates the immune cells (T cells and NK cells) to kill intracellular *M. tuberculosis *bacilli which are usually more or less defiant to drugs. Thus a distinctive role for immunotherapy might be to kill slowly replicating or "dormant" organisms more effectively than current antituberculosis agents, perhaps by administering immunotherapy after the initial phase of treatment. In the present study we evaluated the efficacy only after 4 weeks; this short term immunotherapeutic effect now needs to be corelated in long term experiments.

The reduced systemic Th1 response in tuberculosis [30,31] provides a rationale for using rAg85B as an immunotherapeutic adjunct to treat tuberculosis. We observed a significant shift towards Th1 type of immune response after the immunotherapeutic dosage as there was significant increase in the release of IFNγ and IL-12 with the concomitant abrogation of Th2 response envisaged by the repression in IL-4 levels (Figure 4). This switch from Th2 to Th1 has been observed to be associated with a substantial reduction of pathology in the animals as reported by various groups [5,27,34], thus suggesting that the therapeutic effect of rAg85B may, at least partly, be due to the rectification of immunopathological subversion and the restoration of the Th1/Th2 balance. On the contrary the immune response upon DNA vaccination was in discord of expectations as no antigen specific cytokine production was observed. Even though there exists a lot of uncertainty regarding the immunotherapeutic use of DNA vaccines [35], still we cannot justify the current observation as we did not investigate both for the pathology as well as expression of antigen by DNA vaccine in the affected organs.

## Conclusion

It seems imperative to explore the mechanisms of therapeutic vaccines for future investigations and moreover administration of single antigen induced immunotherapy is expected to alter only one aspect of a complex immune response. An alternative approach will be to generate a multifaceted favourable response. Application of such immunotherapy alone or as an adjunct to conventional chemotherapeutic regimen may result in explicit cure for tuberculosis.

## Materials and methods

### Animals

Specific pathogen-free, 6-8 weeks old female Balb/c mice were obtained from NIPER Mohali, India. Mice were provided with food and water *ad libitum *and the protocol was approved by the institutional animal ethics committee of PGIMER, Chandigarh, India.

### Bacterium culture

*M. tuberculosis *H_37_Rv mantained on LJ medium was inoculated in modified youman's medium and grown as shake culture at 37°C. Bacteria were harvested at mid-log phase and stored at -80°C in aliquots. From one aliquot serially diluted bacterium were inoculated in 7H11 agar medium with OADC at 37°C for 4-5 weeks to count Colony Forming Units (CFUs).

### Recombinant protein and plasmid

Recombinant Ag85B was purified from pET28a clone available in our laboratory. DNA sequence encoding *M. tuberculosis *Ag85B antigen was cloned into pVAX1 (Invitrogen) to yield DNA vaccine pVAX85. The construction and immunogenicity of this DNA vaccine had been previously published in detail [36]. Endotoxin free DNA for vaccination was purified by EndoFree Plasmid Giga kit (QIAGEN), adjusted to a concentration of 1 mg/ml in saline and stored at -20°C until required.

### Animal challenge and immunization / chemotherapy

Mice were infected intravenously via the lateral tail vein with (2 × 10^5 ^CFU/animal) viable *M. tuberculosis *H_37_Rv suspended in 0.1 ml saline. In order to evaluate the protective efficacy and immune responses generated before and after the course of immunotherapy by Ag85B and DNA vaccine based on this antigen and chemotherapy in *M. tuberculosis *infected animals, mice were randomized (5 animals per group) and treated with rAg85B and ATD alone and also in combination. One group of mice received plasmid vaccination based on this protein. Further, to evaluate the effect of immunotherapy on persisters, another group of mice was treated with rAg85B after the completion of short term chemotherapy. The immunization protocol is shown in Figure 1. The mice receiving immunotherapy with rAg85B were administered 10 μg/dose/animal, of protein emulsified in DDA (250 μg/dose/animal). For DNA vaccination 50 μg recombinant plasmid (pVAX85) or empty vector (pVAX1) was injected intramuscularly into quadriceps muscle of each hind leg. The immunization was repeated four times after every week. The control groups were immunized with adjuvant/empty vector alone. The 'short duration' chemotherapy entailed the delivery of 10 mg/kg body weight of isoniazid (INH) and 10 mg/kg body weight of rifampicin (R) (Sigma) daily orally with the help of a gavage from day 14 post infection, for a period of 4 weeks (i.e. 2nd to 6th week) or otherwise stated [37].

### Bacterial counts in organs

Four weeks after treatment completion, the mice were sacrificed and their lungs and spleens were removed. The organs were homogenized in saline containing 0.05% Tween 80. Ten-fold serial dilutions of the homogenates were seeded onto 7H11 agar. The plates were incubated at 37°C for 4 weeks and CFUs were counted after a visible bacterial colony appearance and the result was expressed as log_10 _CFU.

### Cytokine assays

The mice were bled at various time points and blood from each group was pooled to isolate PBMCs by density gradient centrifugation. Single cell suspension was prepared in complete RPMI medium supplemented with 2 mM l-glutamine, 25 mM HEPES buffer, 100 units/ml penicillin, 0.1 mg/ml streptomycin, 1% sodium pyruvate (Sigma), 50 mM 2-mercaptoethanol (Sigma), and 10% fetal calf serum. The lymphocytes (2 × 10^5 ^cells in volume of 100 μl complete RPMI) were cultured in microtiter wells (96-well plates; Nunc) and incubated in quintuplicate with Ag85B (10 μg/ml) and ConA (5 μg/ml) as positive control or medium alone, respectively. The plates were incubated at 37°C in an atmosphere of 5% CO_2 _for 96 hours, and culture supernatants were collected and stored at -20°C until required. IFNγ, IL-12 and IL-4 cytokines were assayed by ELISA kits (Opt EIA™ Set BD Pharmingen, CA, USA) following manufacturer's instructions. Concentrations of cytokines in test samples were determined by comparing absorbances of test samples with absorbances of standards within a linear curve fit. Mean cytokine concentrations (pg/ml) produced in 96-h cultures in response to antigen or mitogen minus concentrations in non-stimulated cultures are presented.

### Statistical analysis

The therapeutic efficacy of different vaccine combinations was compared by Student's *t*-test of variance of the log_10 _CFU and the concentrations of cytokines. A *p *value < 0.05 was considered significant.

## Competing interests

The authors declare that they have no competing interests.

## Authors' contributions

JAS was involved in performing the experiments and analysis of results while GKK and IV participated in the design of experiments, analysis of results and manuscript writing. All authors have read and approved the final manuscript.
